# Maximum power point tracking dataset for a wind energy conversion system based on a reverse-controller for a multilevel boost converter

**DOI:** 10.1016/j.dib.2022.107900

**Published:** 2022-02-02

**Authors:** José Genaro González-Hernández, Rubén Salas-Cabrera, Roberto Vázquez-Bautista, Luis Manuel Ong-de-la-Cruz

**Affiliations:** Tecnológico Nacional de México, Instituto Tecnológico de Ciudad Madero, Tamaulipas. 89440, México

**Keywords:** Wind energy systems, Power point tracking, Control law, Multilevel converters, Real-time control

## Abstract

The database here contains experimental data relevant to an original maximum power point tracking controller for an experimental direct-drive full-variable-speed full-rated converter Type IV Wind Energy Conversion System in standalone operation. The main goal is to maximize power extraction by controlling the duty cycle of a multilevel boost converter, which is responsible for adjusting the angular speed of a permanent magnet synchronous generator coupled to a three-phase induction motor that emulates the wind turbine. Two data acquisition cards with the appropriate signal conditioners were used to obtain measurements of the generator angular speed, output current, and output voltage at the terminals of the multilevel converter. In addition, data related to power coefficient, tip speed ratio, duty cycle, and output power are also included. Two PCs in a Linux real-time platform were used for the emulation, control, and data collection processes. On the other hand, Matlab was used to analyze the data to evaluate the controller's performance to maximize wind power extraction. The database is freely accessible at http://dx.doi.org/10.17632/363d24mcb6.2. This dataset [1] represents a resource for wind power specialists who develop algorithms for wind energy optimization.

## Specifications Table

**Table:** 

Subject	Electrical and Electronic Engineering.
Specific subject area	Renewable Energy, Wind Power Extraction Optimization.
Type of data	Tables of electrical and aerodynamic variables.
How the data were acquired	Experimental measurements performed under specified conditions by using two National Instruments data acquisition cards model PCI6024e, two desktop computers under real-time Linux platform, Hall effect sensors, an encoder and electronic cards to adjust electric signals.
Data format	Raw and analyzed.
Description of data collection	Provide A real-time application interface for Linux was used to acquire the data. The open-source software Scilab/Scicos was programmed to configure data acquisition cards with the help of RTAI-Lib and Xrtailab. Specific software tools were utilized to set the adequate sampling time, adjust the signals, and store the data.
Data source location	Instituto Tecnológico de Ciudad Madero, Ciudad Madero, Tamaulipas, México.
Data accessibility	Repository name: Mendeley Data. Data identification number: DOI:10.17632/363d24mcb6.2. Direct URL to Data: http://dx.doi.org/10.17632/363d24mcb6.2
Related research article	*J.G. González-Hernández, R. Salas-Cabrera, R. Vázquez Bautista, L.M. Ong-de-la-Cruz, J. Rodríguez-Guillén, A novel MPPT PI Discrete reverse-acting controller for a wind energy conversion system, Renewable Energy 178 (2021) 904-915.*https://doi.org/10.1016/j.renene.2021.06.106

## Value of the Data

• This dataset is helpful because it provides insight into maximum power point tracking (MPPT) for wind energy conversion systems (WECS) supported by actual measurements.
• The beneficiaries of this dataset are the professionals working on WECS efficiency and academics interested in wind power extraction optimization.
• These data can be used to 1) support WECS specialists who need raw data for deriving algebraic or differential models that may lead to significant enhancement in the area of wind power optimization; 2) provide an experimental-based framework to WECS professionals working on MPPT techniques for decisions related to wind power efficiency; 3) highlight the actual relationship between tip speed ratio (λ) and power coefficient ($C_{p}$) and their importance in terms of power extraction.
• An additional value of this dataset is that it is one of the first that provide actual measurements relevant to MPPT for WECS.

## 1 Data Description

**Fig. 1:**
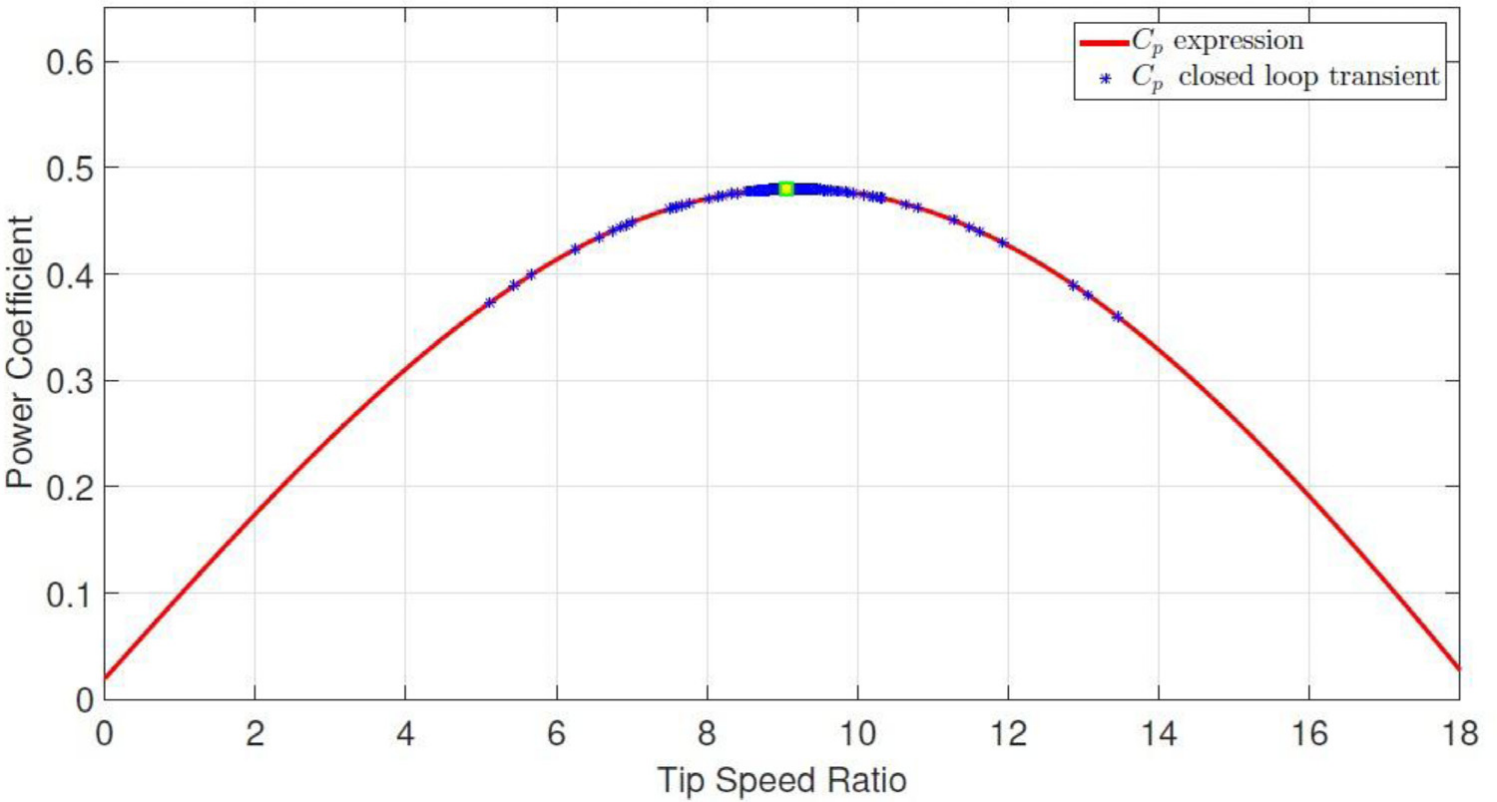
Power Coefficient as a function of the Tip Speed Ratio. Experimental closed-loop operation.

**Fig. 2:**
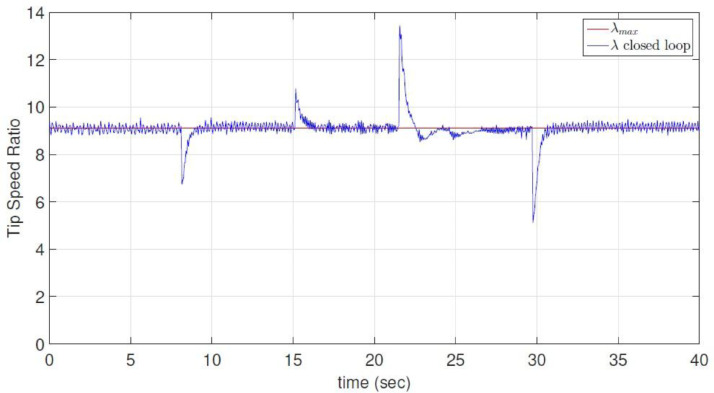
Experimental Tip Speed Ratio. Closed-loop operation.

**Fig. 3:**
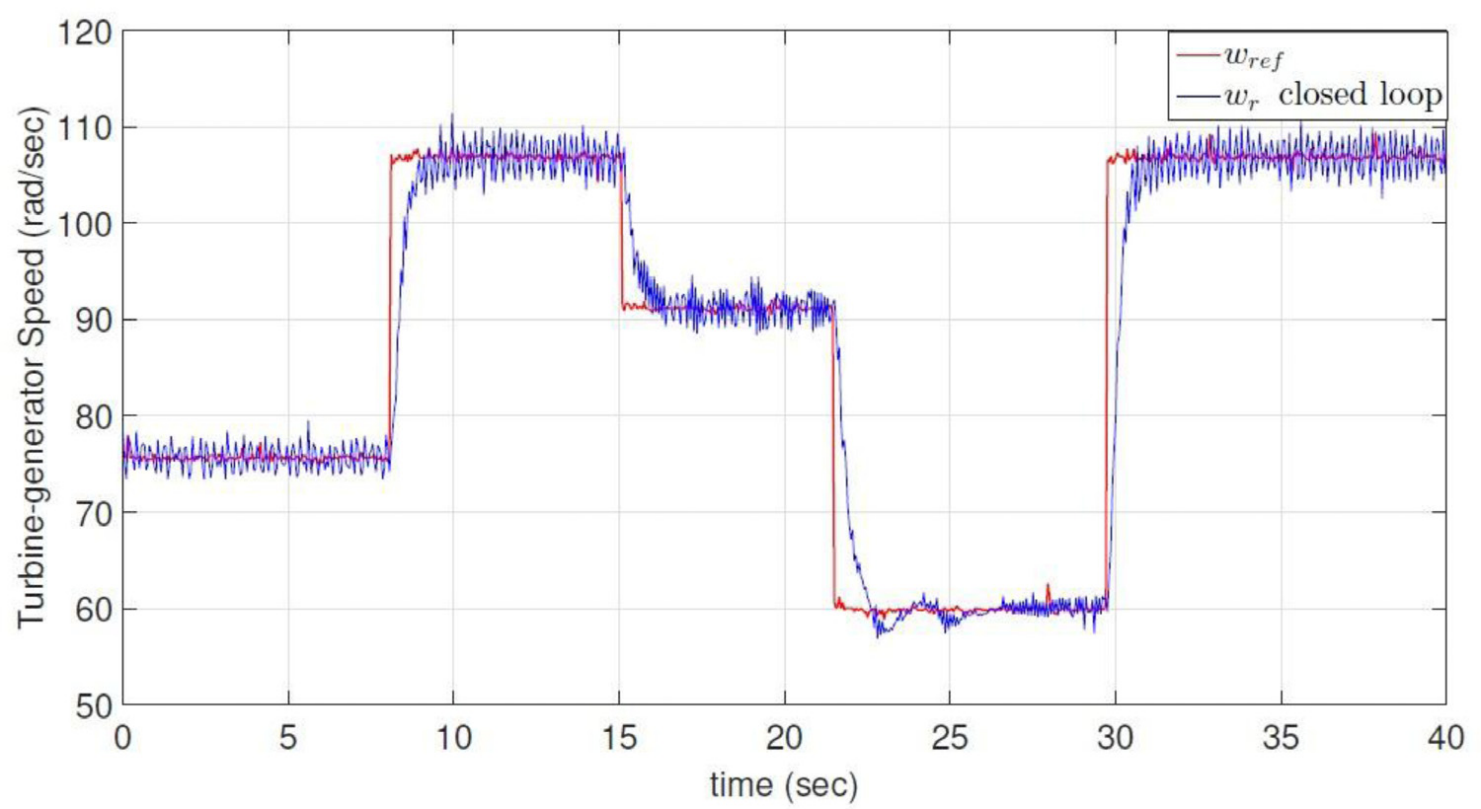
Experimental Turbine-Generator Speed. Closed-loop operation.

The dataset contains five *.dat files that store raw and processed data obtained from the experimental wind energy conversion system using two PCI6424E data acquisition cards and a *.m file (a MATLAB script file) read and plot the *.dat files; in addition, the five *.dat files are also presented in *.csv format. A.dat and B.dat files (Voltage.csv and Current.csv respectively) contain raw data related to the output voltage and current measurements at the terminals of the multilevel boost converter (MBC); C.dat file (Rotor speed.csv) contains the raw data associated with rotor angular speed of the permanent magnet synchronous generator; D.dat (Rotor speed sp.csv) contains the optimum rotor angular speed calculated for different wind speeds of the turbine emulator, and the E.dat file (Duty cycle.csv) represents the duty cycle provided by the discrete-time reverse-acting controller for the MBC. On the other hand, Fig. 1 shows Cp theoretical curve dependent on tip speed ratio and Cp closed-loop transient behavior. Fig. 2 indicates the specific value of the maximum tip speed ratio and its dynamic behavior in closed-loop, and finally, Fig. 3 presents the optimum angular rotor speed before wind changes and also angular rotor speed transient behavior in closed-loop. Measurements were stored for 40 seconds.

## 2 Experimental Design and Methods

**Fig. 4:**
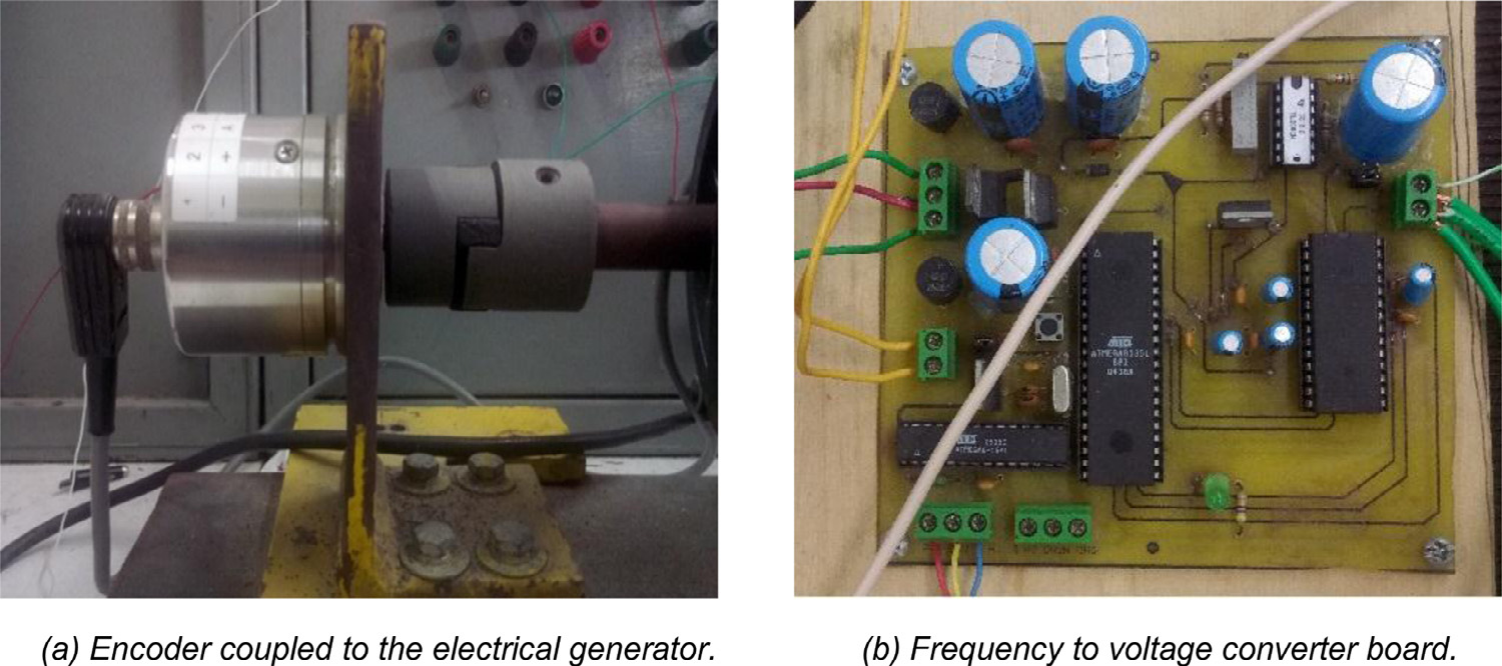
Instrumentation for measuring the rotor speed of the generator.

In order to obtain the data, two PCI6024E data acquisition cards (DAC) installed in two desktop computers were used following all the recommended practices [2], the input channels of these DACs can handle voltages of ±10 volts. Therefore, the first step was to adjust the signals as it is described by [3]. For measuring the angular speed of the generator, an encoder coupled to the rotor shaft was used, as it is shown in Fig. 4a; the encoder converts mechanical rotor speed to frequency according to the relationship 1 rpm = 17.066 Hz.

A custom-made frequency to voltage converter card shown in Fig. 4b, containing two microcontrollers and one digital-analog converter, was employed to obtain an instantaneous voltage that is proportional to the encoder frequency.

An ISO-122 integrated circuit was used to measure the voltage; it contains an isolation amplifier that replicates the input signal at the output terminals.

**Fig. 5:**
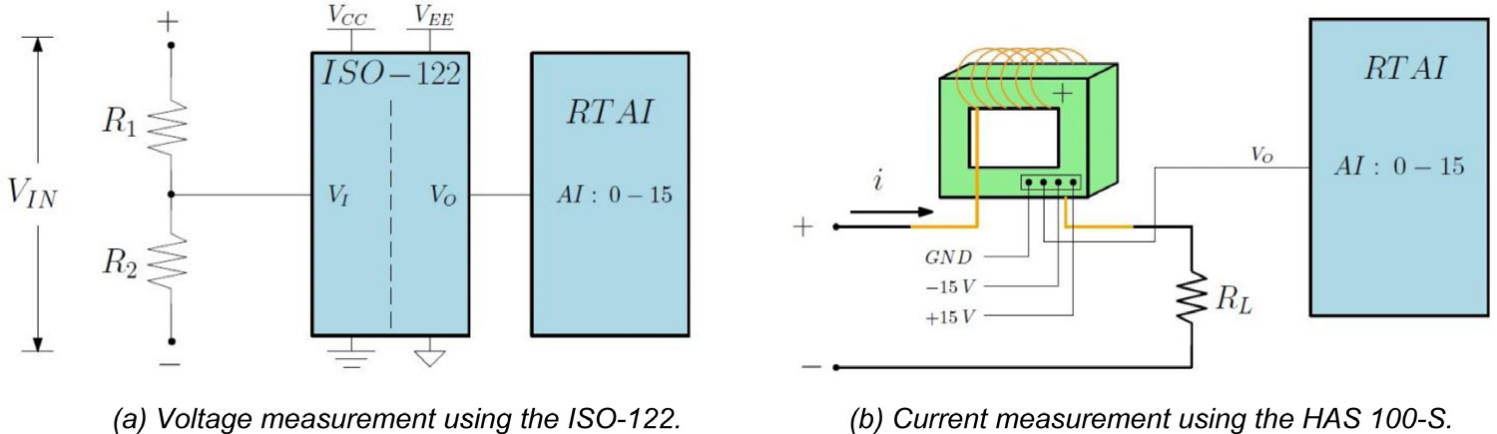
Schematic of the instrumentation for measuring currents and voltages.

This electronic component requires two isolated power supplies to work, one to feed the input and the other to feed the output of the integrated circuit. In addition, the ISO-122 can only handle inputs of ±V_CC_; hence the circuit shown in Fig. 5a was used to keep input voltages within limits. R_1_ = 120 kΩ and R_2_ = 4.7 kΩ were proposed, so V_I_ = 0:037 V_IN_. With this voltage ratio, ±270 V can be measured without saturating the input of the ISO-122 or the hardware of the real-time platform.

For continuous and transient measurement of the current, a Hall effect sensor was used as shown in Fig. 5b. It produces a voltage within ±4 volts depending on the magnitude of the current. Once the real-time platform obtained these signals, they are scaled using mathematical blocks in Scicos/Scilab software as described in [4], so they could be correctly interpreted according to the actual values.

Some works related to Scilab applications are shown in [5,6]. RTAI-Linux was a powerful tool used to support data acquisition and real-time tasks; important information related to this platform can be found in [7], [8].

**Fig. 6:**
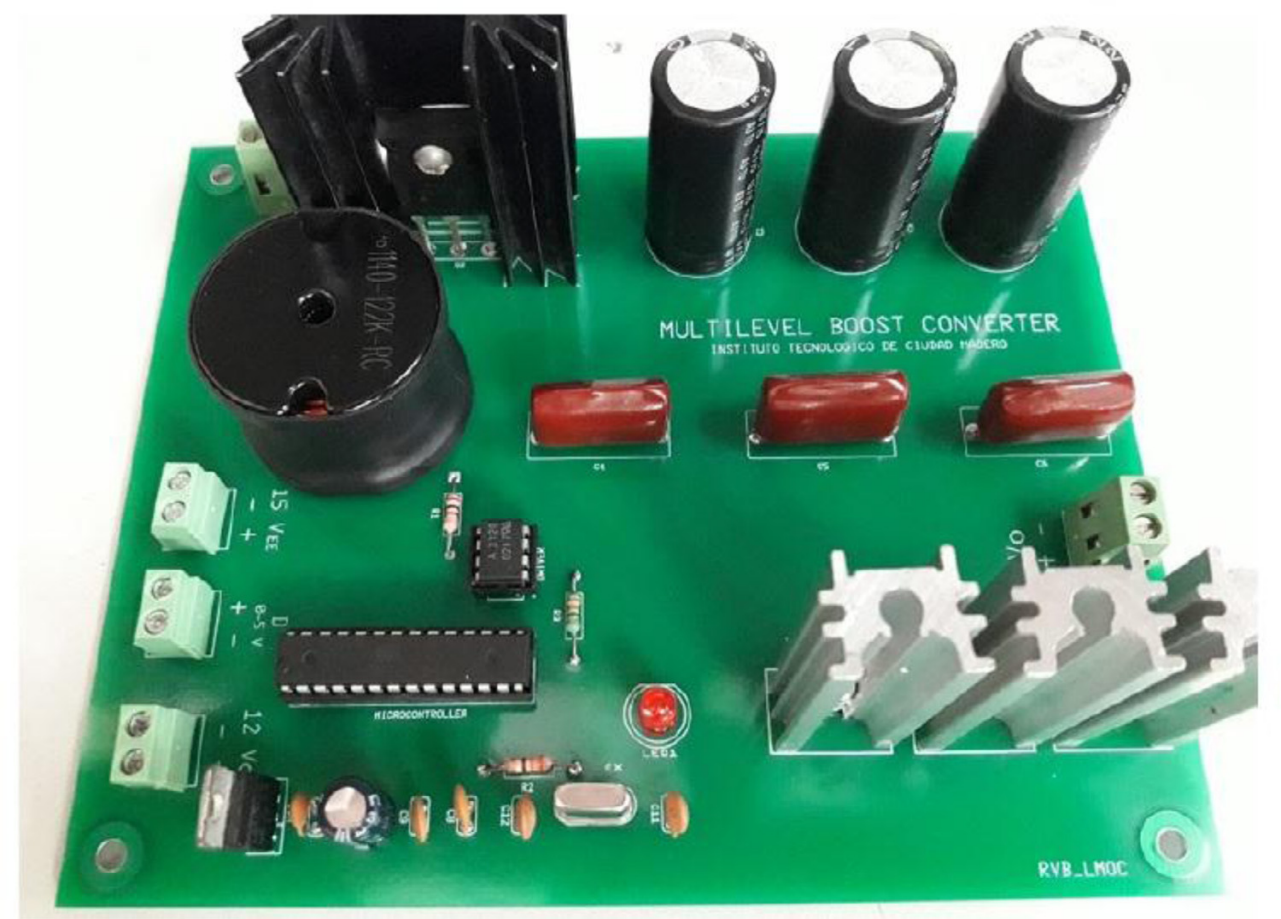
Custom-made Multi-level Boost Converter.

The custom-made MBC is depicted in Fig. 6. Maximum power is extracted from the WECS due to the efficient real-time-based discrete-time reverse-acting controller that defines the duty cycle of this MBC.

## CRediT Author Statement

**José Genaro González-Hernández:** Conceptualization, Formal analysis, Writing – Original Draft, Visualization, Review and editing; **Rubén Salas-Cabrera:** Supervision, Project administration, Methodology, Resources; **Roberto Vázquez-Bautista:** Software, Investigation, Validation; **Luis Manuel Ong-de-la-Cruz:** Software, Investigation, Validation.

## Declaration of Competing Interest

The authors declare that they have no known competing financial interests or personal relationships which have, or could be perceived to have, influenced the work reported in this article.
